# Development of PDAC diagnosis and prognosis evaluation models based on machine learning

**DOI:** 10.1186/s12885-025-13929-z

**Published:** 2025-03-20

**Authors:** Yingqi Xiao, Shixin Sun, Naxin Zheng, Jing Zhao, Xiaohan Li, Jianmin Xu, Haolian Li, Chenran Du, Lijun Zeng, Juling Zhang, Xiuyun Yin, Yuan Huang, Xuemei Yang, Fang Yuan, Xingwang Jia, Boan Li, Bo Li

**Affiliations:** 1 Department of Clinical Laboratory, The Fifth Medical Centre of Chinese PLA General Hospital, Beijing, China; 2 Department of Clinical Laboratory, Beijing Electric Power Teaching Hospital, Capital Medical University, Beijing, China

**Keywords:** Pancreatic ductal adenocarcinoma, Machine learning, DeepSurv, Prognosis prediction, Individualized treatment recommendation

## Abstract

**Background:**

Pancreatic ductal adenocarcinoma (PDAC) is difficult to detect early and highly aggressive, often leading to poor patient prognosis. Existing serum biomarkers like CA19-9 are limited in early diagnosis, failing to meet clinical needs. Machine learning (ML)/deep learning (DL) technologies have shown great potential in biomedicine. This study aims to establish PDAC differential diagnosis and prognosis assessment models using ML combined with serum biomarkers for early diagnosis, risk stratification, and personalized treatment recommendations, improving early diagnosis rates and patient survival.

**Methods:**

The study included serum biomarker data and prognosis information from 117 PDAC patients. ML models (Random Forest (RF), Neural Network (NNET), Support Vector Machine (SVM), and Gradient Boosting Machine (GBM)) were used for differential diagnosis, evaluated by accuracy, Kappa test, ROC curve, sensitivity, and specificity. COX proportional hazards model and DeepSurv DL model predicted survival risk, compared by C-index and Log-rank test. Based on DeepSurv’s risk predictions, personalized treatment recommendations were made and their effectiveness assessed.

**Results:**

Effective PDAC diagnosis and prognosis models were built using ML. The validation set data shows that the accuracy of the RF, NNET, SVM, and GBM models are 84.21%, 84.21%, 76.97%, and 83.55%; the sensitivity are 91.26%, 90.29%, 89.32%, and 88.35%; and the specificity are 69.39%, 71.43%, 51.02%, and 73.47%. The Kappa values are 0.6266, 0.6307, 0.4336, and 0.6215; and the AUC are 0.889, 0.8488, 0.8488, and 0.8704, respectively. BCAT1, AMY, and CA12-5 were selected as modeling parameters for the prognosis model using COX regression. DeepSurv outperformed the COX model on both training and validation sets, with C-indexes of 0.738 and 0.724, respectively. The Kaplan-Meier survival curves indicate that personalized treatment recommendations based on DeepSurv can help patients achieve survival benefits.

**Conclusion:**

This study built efficient PDAC diagnosis and prognosis models using ML, improving early diagnosis rates and prognosis accuracy. The DeepSurv model excelled in prognosis prediction and successfully guided personalized treatment recommendations and supporting PDAC clinical management.

## Background

Pancreatic cancer (PC) is one of the most common malignancies of the digestive tract, characterized by high aggressiveness and poor prognosis. According to the latest research data from the Surveillance Research Center of the American Cancer Society, the estimated number of new cases of pancreatic cancer in 2024 is 66,400, with an estimated 51,800 deaths [1]. The annual growth rate of pancreatic cancer currently stands at 0.5–1.0%, and it is projected to become the second leading cause of cancer deaths in Western countries by 2030 [2]. Among pancreatic cancers, pancreatic ductal adenocarcinomas (PDAC), originating from the pancreatic ductal epithelial cells of the exocrine pancreas, account for approximately 90% of all pathological types and are highly aggressive, with a 5-year survival rate of only 10% and a 10-year survival rate as low as 1% [3]. PDAC often have no obvious symptoms in the early stages, leading to most patients missing the opportunity for early surgical intervention. Among the serum biomarkers for diagnosing PDAC, CA19-9 is the most widely used and holds the highest value. However, its sensitivity in diagnosing PDAC is insufficient for achieving early diagnosis of the disease. Therefore, combining multiple serological biomarkers to enhance the sensitivity of PDAC diagnostic markers is an effective approach for achieving early diagnosis of PDAC [4, 5].

Artificial intelligence technology represents an emerging approach in medical research, with its promising development prospects spanning various fields such as imaging diagnosis, pathological microscopic image recognition, clinical laboratory data mining, precision surgery, and personalized medicine. With advancements in structural analysis and sequencing technologies, clinical and biological information from tumor patients has been growing exponentially, posing immense challenges in analyzing correlations and attributing causes among these vast data. In recent years, significant improvements in computer processing speeds have drastically enhanced large-scale parallel computing and matrix operations. The advantages of machine learning/deep learning have gradually emerged, particularly in the biomedical and clinical medicine domains, where they have achieved remarkable outcomes. The utilization of machine learning/deep learning methods for diagnosing various tumors, conducting disease diagnosis, and assessing risk has gained widespread recognition. Therefore, leveraging deep learning methods based on clinical data from tumor patients to predict, diagnose, evaluate patients’ clinical status, and guide clinicians in treatment is a reliable and efficient research strategy.

In the realm of medical research, the Cox proportional hazards model is frequently employed to evaluate the significance of covariates related to patient prognosis in events such as mortality or recurrence of malignancies, thereby informing the selection of therapeutic strategies for patients. However, the Cox model presupposes a linear relationship between covariates and risk factors, which may not adequately encapsulate the complexity of risk functions observed in real-world datasets. Consequently, more sophisticated survival analysis models are necessary in clinical practice to effectively model survival data with nonlinear risk functions. Jared Katzman introduced a deep learning algorithm based on the Faraggi-Simon network, termed DeepSurv, which has demonstrated superior predictive performance and flexibility compared to the Cox proportional hazards model in medical applications [6].

This study aims to achieve two objectives. Firstly, by leveraging traditional machine learning techniques and incorporating commonly used clinical serum biochemical indicators and tumor markers, we aim to establish a machine learning-based differential diagnostic model for PDAC. This model is expected to differentiate PDAC from other gastrointestinal malignancies and normal non-tumor individuals. Secondly, we plan to utilize the open-source Python code available on Github to construct a DeepSurv risk score model for predicting the prognosis of PDAC patients. Through this model, we aim to stratify patients into risk groups and match them with individualized treatment plans.

## Methods

### Data sources

A total of 117 patients with PDAC admitted to the Fifth Medical Center of Chinese PLA General Hospital from September 2022 to October 2023 were collected through the electronic medical record system. These patients ranged in age from 38 to 84 years, with a mean age of 60.61 years. Among them, 82 were male and 35 were female, resulting in a male-to-female ratio of 2.34:1. Inclusion criteria encompassed: (1) patients with PDAC confirmed by pathological biopsy of pancreatic tissue; (2) patients who were first diagnosed and admitted to our hospital for treatment. Exclusion criteria were: (1) patients with metastatic pancreatic cancer; (2) those with coexisting infectious diseases or digestive system disorders, such as viral hepatitis, acute or chronic pancreatitis, and cholecystitis; (3) patients with other pathological types of pancreatic cancer or other types of malignancies; (4) patients with hematological diseases; (5) diabetic patients; (6) patients with insufficient, contaminated, or lipemic serum samples.

To enrich the dataset, subsequent serum sample test data from these patients were also included, totaling 345 samples. To enhance the generalization capability of the models, control group data comprising other gastrointestinal tumors, pancreatitis, and healthy controls were incorporated, including 40 cases each of gastric cancer, liver cancer, and colorectal cancer patients, 8 cases of chronic pancreatitis patients, and 50 healthy individuals, collectively forming the non-PDAC group with a total of 178 cases. This resulted in a total of 523 serum samples for both the PDAC and non-PDAC groups. No statistically significant differences in age or gender were observed between the groups (*p* > 0.05). These case and control groups were primarily utilized for the development and validation of machine learning-based diagnostic models.

All patients were followed up for 12 months to record survival status (deceased, alive, lost to follow-up), enabling the calculation of overall survival (OS) time. Follow-up was conducted via telephone interviews with patients or their relatives, and death dates recorded in the electronic medical record system were used when available. Information on treatment regimens received by patients was also recorded, serving as a data source for individualized treatment recommendations within the models. Follow-up data were primarily employed for the establishment and validation of prognostic models and treatment recommendation models. All research participants have signed informed consent forms, and this study has been approved by the Ethics Committee of the Fifth Medical Center of Chinese PLA General Hospital (Ethics Approval Number: KY-2024-4-63-1).

### Study indicators

In our previous research, we identified Branched-chain Amino Acid Transaminase 1 (BCAT1) as a potential serological diagnostic biomarker for PDAC. Consequently, BCAT1 was included as a diagnostic parameter in this study. We utilized ELISA kits from EIAab to measure serum BCAT1 concentrations. Based on previously published literature, we selected 11 biochemical indicators and tumor markers that are routinely tested in clinical practice as the research indicators [7–9], including glucose (GLU), alanine aminotransferase (ALT), total bilirubin (TBiL), direct bilirubin (DBiL), alkaline phosphatase (ALP), aspartate aminotransferase (AST), amylase (AMY), carbohydrate antigen 19-9 (CA19-9), carbohydrate antigen 12-5 (CA12-5), carcinoembryonic antigen (CEA), and carbohydrate antigen 72-4 (CA72-4). Biochemical markers were analyzed using a Hitachi 008AS fully automatic biochemical analyzer, while tumor markers were detected using the Roche cobas® 8000 modular analyzer series and its corresponding reagents.

### Establishment of machine learning diagnostic models for PDAC

In this study, data processing and analysis were performed using R software (version 4.3.1) with the caret package (version 6.0–94). Four supervised learning algorithms were utilized: Random Forests (RF), Neural Network (NNET), Support Vector Machines (SVM), and Gradient Boosting Machine (GBM). Model performance was evaluated using accuracy, Kappa statistic, ROC curves, sensitivity, and specificity. The methodology and steps involved in the modeling process include: (1) Data preprocessing and splitting were first performed. The preProcess() function was utilized to handle missing values, and the createDataPartition() function was employed to divide the entire dataset into a test set and a training set in a 7:3 ratio, respectively, for sample training and validation. (2) The rfe() function was applied for feature selection to incorporate optimal model parameters. (3) The train() function was utilized to establish the diagnostic model using the training set samples, followed by resampling for preliminary model evaluation. (4) The confusionMatrix() function was employed to further validate and evaluate the model using the sample validation set data. All the functions mentioned above are derived from the caret package.

### Establishment of PDAC risk prediction models

#### Indicator selection

The aforementioned 12 serum biochemical and tumor marker indicators were included and subjected to regression analysis using the R survival package. Univariate COX regression analysis was performed to initially screen prognostic modeling indicators, with *p* < 0.05 as the selection criterion. Subsequently, multivariate COX regression analysis was conducted to further screen the initially selected indicators, again using *p* < 0.05 as the criterion. The finally selected indicators were used as input layer indicators for the risk prognosis model and employed in modeling.

#### Establishment of COX proportional hazards model and deep learning deepsurv model

**Fig. 1:**
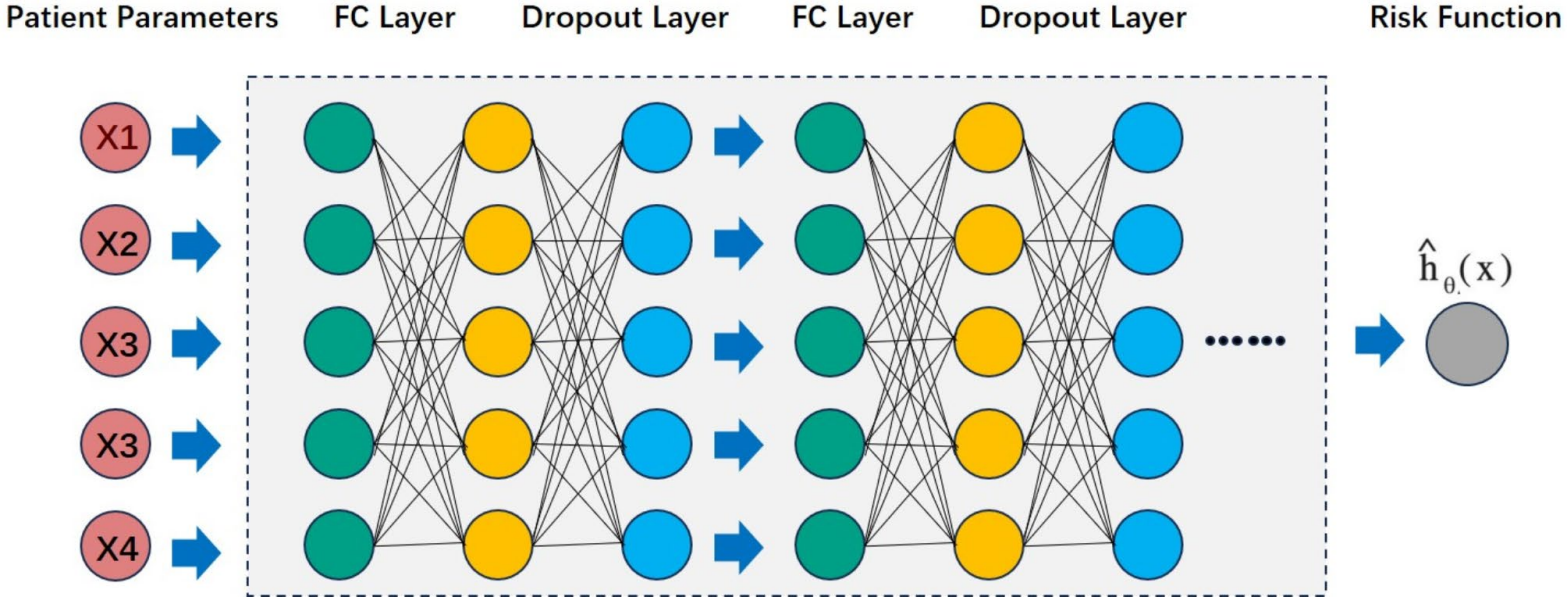
Network Connection Schema of DeepSurv

Using the screened indicators as input variables, all data from the PDAC group were randomly divided into a training group and a validation group in a 1:1 ratio using the sklearn library in Python. To compare the predictive performance of linear prediction models with deep learning models, this study established a COX proportional hazards model using lifelines and built a deep learning model by downloading the source code of the Deepsurv library from Github (https://github.com/jaredleekatzman/DeepSurv). The C-index and Log-rank test were used to evaluate the predictive performance of both models, and Kaplan-Meier curves were plotted to visually demonstrate their performance. The parameter settings for the Deepsurv model were as follows, with the parameters adopted being the preset ones from the original code: number of layers (n_layers) = 1; number of nodes per layer (n_nodes) = 10; activation function = selu; learning rate = 0.001; decay rate = 5.667e-4; momentum = 0.887; L2 regularization (l2_reg) = 6.551; dropout = 0.661; optimizer = nadam. The training iteration was set to 1000 cycles. The Deepsurv network structure (Fig. 1) and algorithm are illustrated below, where Xn represents the input features. The network comprises fully connected layers and Dropout layers, and the network output h0(x) represents the predicted log-hazard function. The DeepSurv model employs the Partial Log-Likelihood (PLL) based on the Cox proportional hazards model as its loss function. During model training, the training loss function and C-index are monitored as convergence criteria to ensure that the model exhibits good generalization performance on the validation set.

$$\:l\left( {\theta \:} \right): = - \frac{1}{{{N_{E = 1}}}}\sum\limits_{i:{E_i} = 1} {\left( {{{\hat h}_{\theta \:}}\left( {{x_i}} \right) - log\sum\limits_{j \in \:\Re \left( {{T_i}} \right)} {{e^{{{\hat h}_\theta }\left( {{x_j}} \right)}}} } \right) + \lambda \: \cdot \:\left\| \theta \right\|_2^2} $$

### Evaluation of treatment recommendation function

The Deepsurv model stratifies patient datasets into distinct risk groups to facilitate personalized treatment matching and recommendation. By leveraging the risk scores calculated by the model, it predicts whether patients can derive clinical survival benefits from various individualized treatment regimens. In this study, we employed this scheme to make individualized treatment recommendations for three different PDAC treatment approaches using survival data. These include, in addition to conventional chemotherapy, (1) targeted therapy with drugs such as nimotuzumab, donafenib, bevacizumab, anlotinib, lenvatinib, and surufatinib; (2) PD-1 tumor immunotherapy with drugs like tislelizumab, sintilimab, envafolimab, camrelizumab, durvalumab, toripalimab, and nivolumab; and (3) surgical treatment encompassing four guideline-recommended procedures: pancreaticoduodenectomy, distal pancreatectomy with splenectomy, total pancreatectomy, and minimally invasive radical pancreatic cancer resection. Kaplan-Meier survival curves were utilized to assess whether the Deepsurv individualized treatment recommendation system can aid patients in achieving survival benefits. The parameter settings for the Deepsurv model were the same as those used in the prognosis model.

## Results

### Establishment of a machine learning diagnostic models for PDAC

#### Data processing and feature selection

For the obtained raw dataset, we employed R to perform missing value handling, feature selection, data splitting, and other steps, ultimately yielding a complete dataset suitable for modeling. Firstly, predictive mean matching (PMM) was adopted to impute the missing values. Subsequently, recursive feature elimination (RFE) was applied for feature selection. The results indicated that the PDAC machine learning diagnostic model achieved the highest accuracy with 11 variables (ALT was excluded), including CA19-9, AMY, CA12-5, CEA, TBiL, GLU, DBiL, BCAT1, ALP, CA72-4, and AST.

#### Data splitting

The dataset was divided into a training set and a validation set in a 7:3 ratio using the createDataPartition() function through random stratified sampling. A total of 523 cases, including PDAC (345 cases) and non-PDAC (178 cases), were included, with 336 cases [(523 × 70) ÷ 100 = 366.1, approximately 366] as the training set and 157 cases as the validation set. No significant differences were observed between the training group and the validation group in demographic characteristics such as gender and age (*P* > 0.05).

#### Hyperparameter tuning

**Fig. 2:**
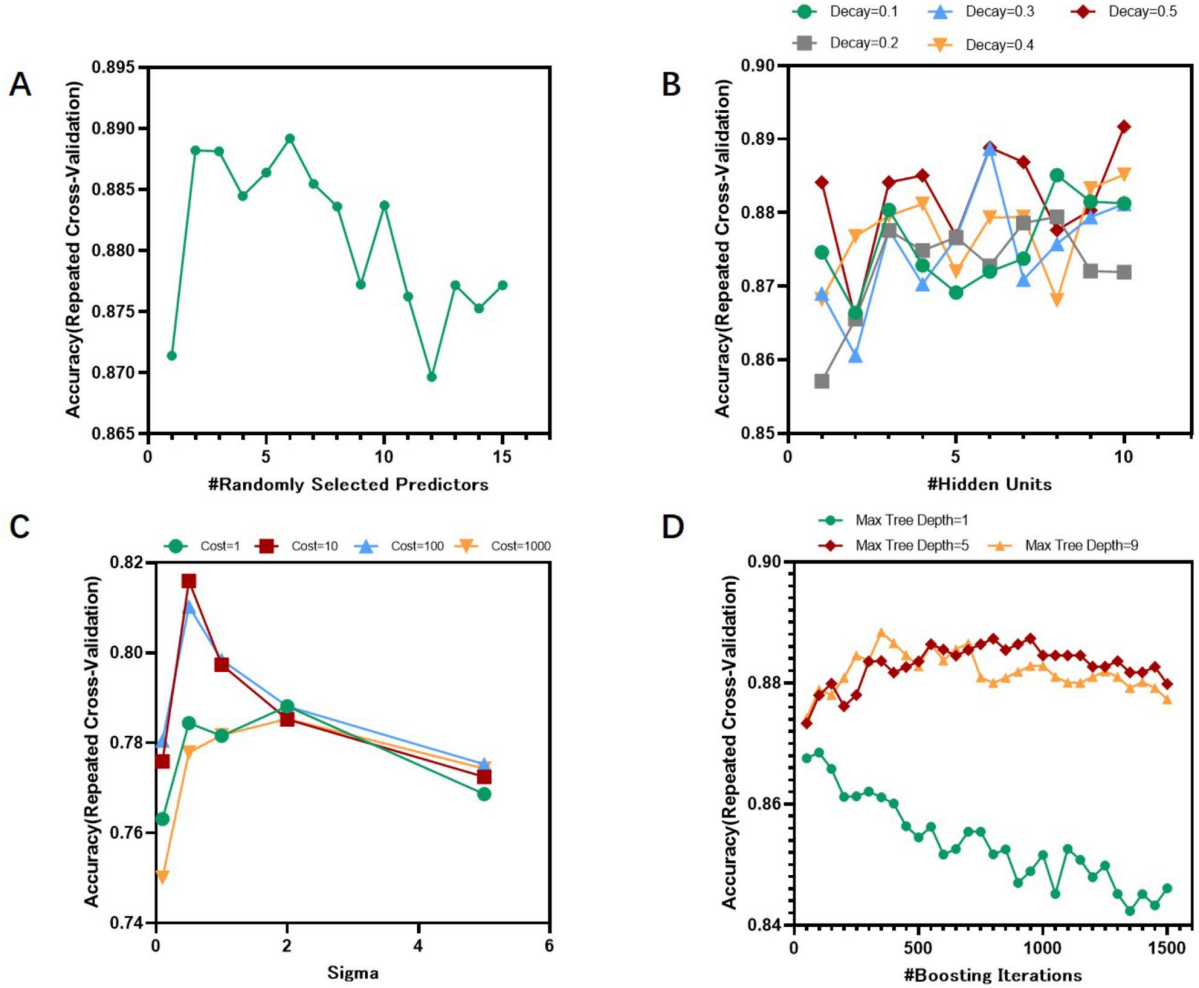
Line charts of accuracy for four models under different parameter values. (**A**) The accuracy of the RF model reaches the peak when the mtry value is 6. (**B**) The accuracy of the NNET model reaches the peak when the size is 10 and the decay is 0.5. (**C**) The accuracy of the SVM model reaches the peak when the sigma is 0.5 and the C is 10. (**D**) The accuracy of the GBM model reaches the peak when the n.trees is 400 and the interaction.depth is 9

To optimize the diagnostic performance of the aforementioned models, we employed grid search tuning to adjust the hyperparameters of the RF, NNET, SVM, and GBM models. During the hyperparameter tuning process of the model, we used 10-fold cross-validation to prevent overfitting and ensure the robustness of model evaluation. The methods and parameter selection for hyperparameter tuning were referenced from the official caret documentation published on GitHub: https://topepo.github.io/caret/model-training-and-tuning.html. As depicted in Fig. 2; Table 1, for the RF model, models were constructed with mtry values ranging from 1 to 15, and highest accuracy was achieved when mtry was set to 6. In the NNET model, the parameters size and decay were set to seq(1, 10, 1) and seq(0.1, 0.5, 0.1) respectively, with highest accuracy observed at size = 10 and decay = 0.5. For the SVM model, the parameters sigma and C were set to [0.1, 0.5, 1, 2, 5] and [1, 10, 100, 1000] respectively, yielding highest accuracy when sigma = 0.5 and C = 10. Lastly, in the GBM model, the parameters n.trees, interaction.depth, shrinkage, and n.minobsinnode were adjusted, with n.trees ranging from (1:30)*50, interaction.depth set to c(1, 5, 9), shrinkage fixed at 0.1, and n.minobsinnode at 20. Highest accuracy was achieved when n.trees was 400 and interaction.depth was 9.

**Table 1:** Hyperparameter optimization

Models	Parameters	Value Range	Optimal Parameters
RF	mtry	mtry = c(1:15)	6
NNET	size	size = seq(1, 10, 1)	10
	decay	decay = seq(0.1, 0.5, 0.1)	0.5
SVM Radial	sigma	sigma =[0.1,0.5,1,2,5]	0.5
	C	C=[ 1, 10, 100, 1000]	10
GBM	n.trees	n.trees = (1:30)*50	400
	interaction.depth	interaction.depth = c(1, 5, 9)	9
	shrinkage	shrinkage = 0.1	0.1
	n.minobsinnode	n.minobsinnode = 20	20

#### Model establishment, validation, and evaluation of diagnostic experiments

**Fig. 3:**
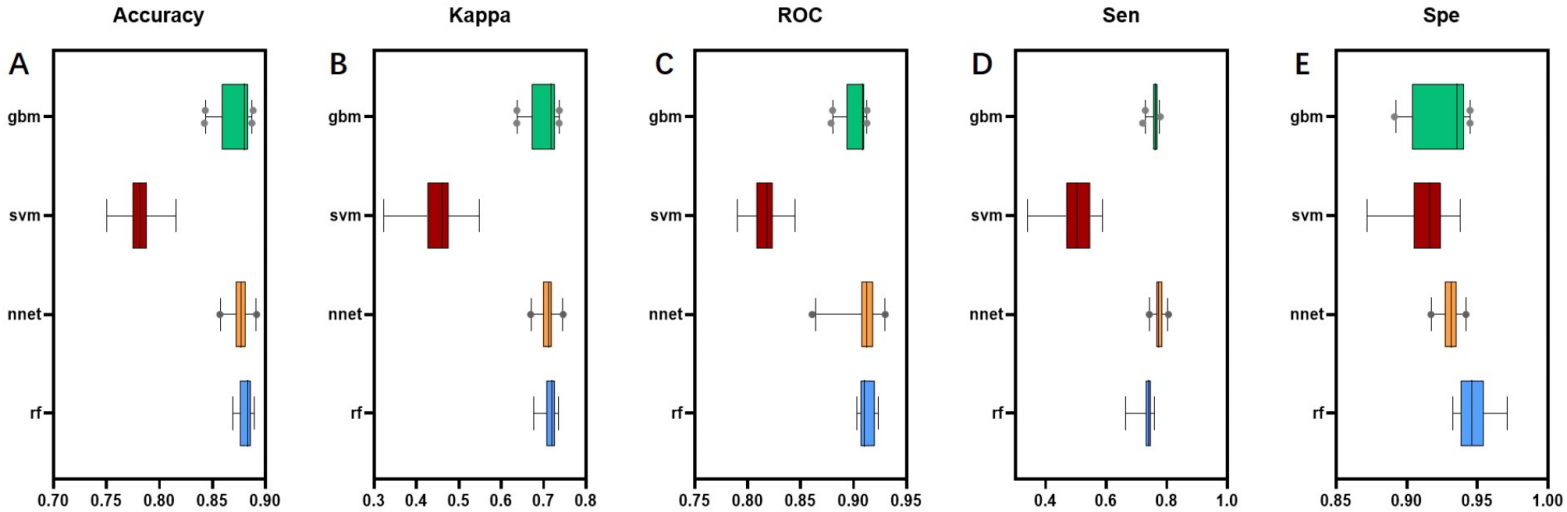
Boxplot comparing the bootstrap resampling performance of four models. (**A**) to (**E**) represent the Accuracy, Kappa Value, ROC, Sensitivity, and Specificity of the four machine learning models respectively

Bootstrap resampling was utilized to resample the training set data, using 10-fold cross-validation and repeating the sampling process 30 times. Boxplots and matrix scatterplots were employed to evaluate the resampling results. As shown in Fig. 3, the SVM model performed inferior to the other three models in terms of accuracy, consistency, AUC, sensitivity, and specificity. The highest accuracy achieved by the four models was 97.2%, with a median of 90.2%; the highest Kappa consistency was 0.939, with a median of 0.768; the highest AUC was 1.00, with a median of 0.945; the highest sensitivity was 100%, with a median of 81.8%; and the highest specificity was 100%, with a median of 91.8%.

**Fig. 4:**
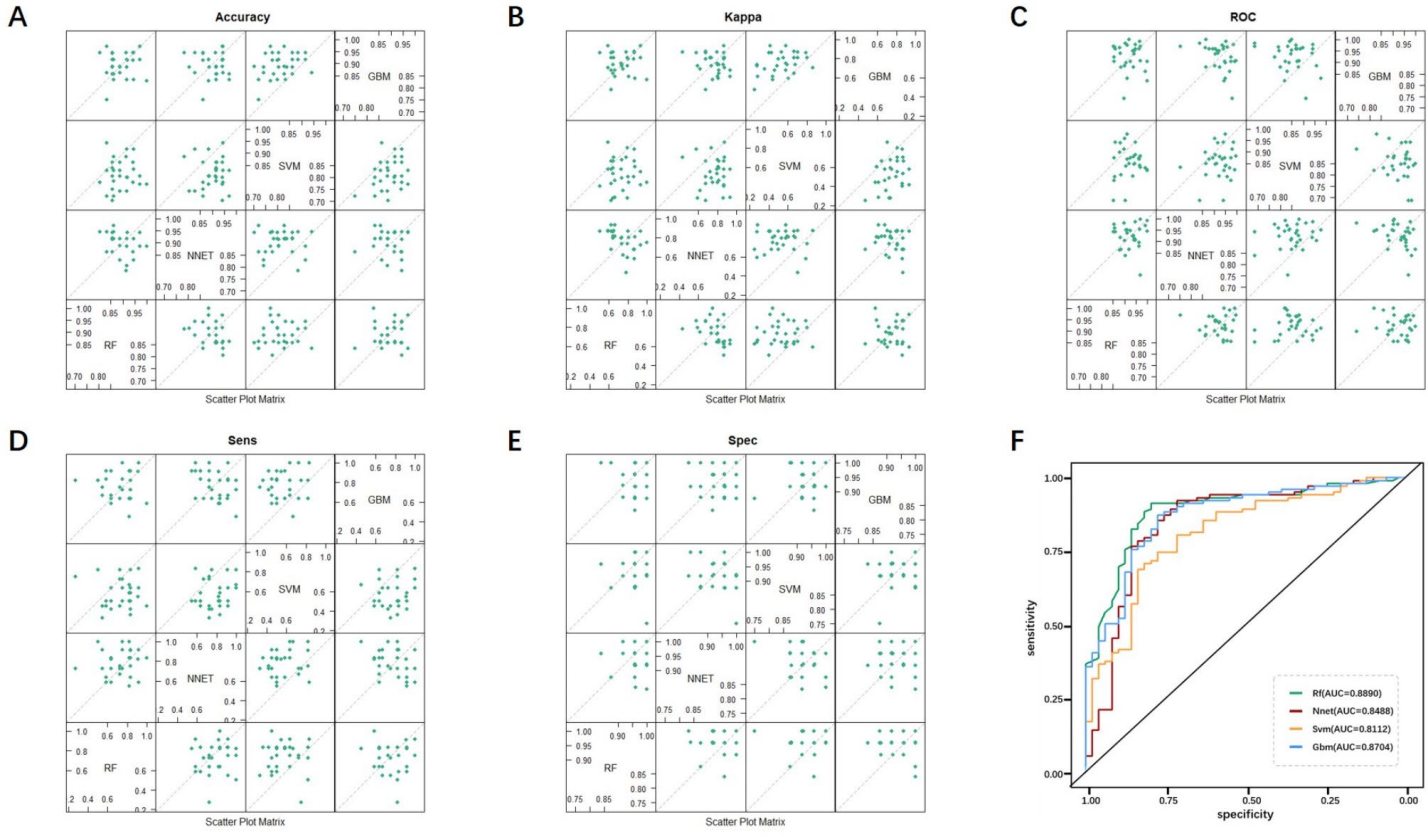
Performance validation and diagnostic experiment evaluation of four models. (**A**) to (**E**) are matrix scatter plots comparing the performance of the four machine learning models, representing Accuracy, Kappa Value, Sensitivity, Specificity, and ROC respectively. (**F**) ROC curves of the four machine learning models, with RF model having the largest AUC, followed by GBM, NNET, and SVM

Validation was conducted using the validation set data, revealing accuracy rates of 84.21%, 84.21%, 76.97%, and 83.55% for the RF, NNET, SVM, and GBM models respectively; sensitivity rates of 91.26%, 90.29%, 89.32%, and 88.35%; and specificity rates of 69.39%, 71.43%, 51.02%, and 73.47%. Notably, the RF and NNET models achieved the highest accuracy, the RF model demonstrated the best sensitivity, and the GBM model showed the highest specificity. Using pathological reports as the gold standard, the diagnostic performance of the four models was evaluated through ROC curves, with the AUC and its 95% confidence interval calculated. Additionally, Kappa consistency tests were performed to assess the agreement between model diagnoses and the gold standard, yielding Kappa values of 0.6266, 0.6307, 0.4336, and 0.6215 for the RF, NNET, SVM, and GBM models respectively; and AUCs of 0.889, 0.8488, 0.8488, and 0.8704. Among them, the NNET model showed the best consistency with the gold standard, while the RF model had the highest AUC. These results are summarized in Table 2; Fig. 4.

**Table 2:** Evaluation of validation and diagnostic experiments for four models in the validation set

Models	Accuracy	Kappa	Sensitivity	Specificity	AUC
RF	84.21%	0.6266	91.26%	69.39%	0.889
NNET	84.21%	0.6307	90.29%	71.43%	0.8488
SVM	76.97%	0.4336	89.32%	51.02%	0.8488
GBM	83.55%	0.6215	88.35%	73.47%	0.8704

### Establishment of COX proportional hazards prognostic model and deep learning deepsurv model

#### Screening of modeling indicators

A total of 12 indicators, including BCAT1, GLU, ALT, TBil, DBil, ALP, AST, AMY, CEA, CA19-9, CA72-4, and CA12-5, were included for screening. Through univariate COX regression analysis, seven indicators (BCAT1, ALT, AST, AMY, CEA, CA19-9, and CA12-5) were selected based on a significance level of *p* < 0.05 (Table 3). Subsequently, multivariate COX regression analysis was performed to further screen the seven indicators included in the model. Based on a significance level of *p* < 0.05, BCAT1, AMY, and CA12-5 were ultimately selected as input layer indicators for modeling the risk prognostic model (Table 4).

**Table 3:** Indicators screened by univariate regression analysis

id	HR	HR.95 L	HR.95 H	*p* value
BCAT1	1.222568112	1.170075606	1.277415563	2.84E-19
GLU	1.009256645	0.965878317	1.054583127	0.681015859
ALT	1.002930906	1.001202051	1.004662745	0.000885141
TBiL	1.003119907	0.999728405	1.006522914	0.071425421
DBiL	1.003284165	0.998982189	1.007604666	0.134786127
ALP	1.000200365	0.999760254	1.00064067	0.37229346
AST	1.004464273	1.002561743	1.006370413	4.13E-06
AMY	0.984608485	0.977889616	0.991373518	9.00E-06
CEA	1.001051625	1.000163977	1.001940061	0.020220983
CA19-9	1.000056789	1.000038761	1.000074818	6.66E-10
CA72-4	0.999877564	0.998369951	1.001387453	0.873635535
CA12-5	1.001398536	1.000980446	1.001816802	5.40E-11

**Table 4:** Indicators screened by multivariate regression analysis

id	HR	L95CI	H95CI	*p* value
BCAT1	1.228861	1.172341	1.288107	9.61E-18
ALT	0.997963	0.992627	1.003328	0.45606
AST	1.005093	0.998935	1.011289	0.105204
AMY	0.985601	0.978518	0.992735	8.09E-05
CEA	1.000073	0.998758	1.00139	0.91346
CA19-9	1.000012	0.999982	1.000041	0.436868
CA12-5	1.000886	1.000253	1.001519	0.006038

#### Model establishment and validation

**Fig. 5:**
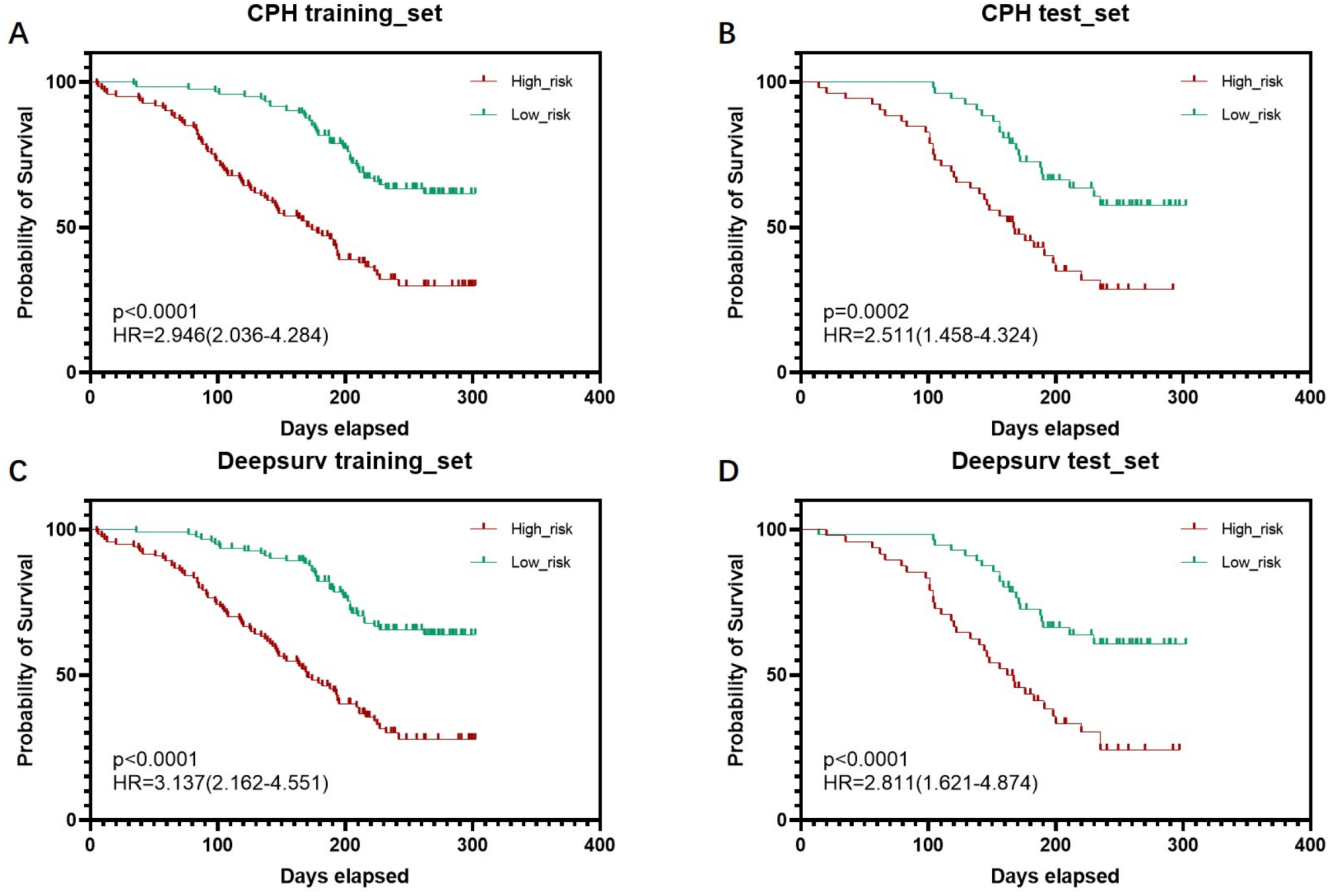
Evaluation of COX proportional hazards prognostic model and deep learning Deepsurv model. (**A**) to (**B**) are the Kaplan-Meier survival curves of the CPH training group and the CPH validation group, while (**C**) to (**D**) are the Kaplan-Meier survival curves of the Deepsurv model training group and the Deepsurv validation group. The High risk group represents patients predicted as high-risk by the model, while the Low risk group represents patients predicted as low-risk by the model

Using BCAT1, AMY, and CA12-5, which were identified through both univariate and multivariate COX regression analyses, as input variables, all data were randomly split into training and validation sets in a 1:1 ratio using the sklearn library in Python, with stratified sampling employed to ensure that the distribution of categories remained consistent. The Cox proportional-hazards model (CPH) was established using the lifelines library, yielding the following results: the C-index (Concordance Index) for the CPH training set was 0.722, with a Log-rank test *p*-value < 0.0001; in the validation set, the C-index was 0.703, with a Log-rank test *p*-value < 0.0001. Additionally, the deep learning Deepsurv model was established using the Deepsurv library, revealing a C-index of 0.738 for the training set, with a Log-rank test *p*-value < 0.0001; in the validation set, the C-index was 0.724, with a Log-rank test *p*-value < 0.0001 (Table 5). Both models demonstrated excellent value in assessing PDAC risk prognosis, with the Deepsurv model performing slightly better. We calculated the predicted risk score (partial hazard) for each sample using the CoxPHFitter model and the Deepsurv model, respectively. Samples with a risk score above the median were classified as the high-risk group, while those with a risk score below the median were classified as the low-risk group. Kaplan-Meier curves were then plotted for the two groups to evaluate the risk prediction ability of the models. Kaplan-Meier survival curves showing that both the CPH and Deepsurv models predicted poorer overall prognosis for high-risk patients and better overall prognosis for low-risk patients. Both models also performed well in the validation sets (*p* < 0.001) (Fig. 5), indicating good agreement between the predicted and actual patient prognoses.

**Table 5:** Concordance index and Log-rank test *p*-values for the training and validation groups of the CPH and deepsurv models

Models	CPH		Deepsurv	
	C-index	Log-rank p Value	C-index	Log-rank p Value
train_group	0.722259035	3.35E-09	0.7381365	7.31E-10
test_group	0.703409382	6.52E-04	0.7236606	1.14E-04

### Evaluation of treatment recommendation capability of the deepsurv models

**Fig. 6:**
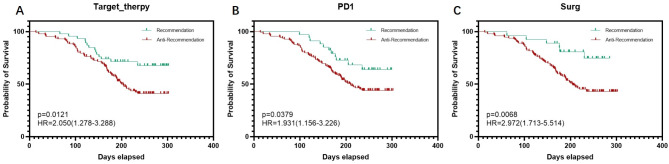
Performance validation of the treatment recommendation system in the Deepsurv library. (**A**) to (**C**) show the Kaplan-Meier survival curves for patients who did or did not receive the system’s recommended targeted drug therapy, PD-1 cancer immunotherapy, and surgical treatment, respectively. The recommendation group represents the patient group that received treatment consistent with the system’s recommendations, while the anti-recommendation group represents the patient group that received treatment inconsistent with the system’s recommendations

Using the sklearn library in Python, all data were randomly split into training and validation sets in a 1:1 ratio. The treatment recommendation system within the Deepsurv library was employed to provide individualized treatment recommendations for three treatment options: targeted drug therapy, PD-1 immunotherapy, and surgical treatment. Kaplan-Meier survival curves were utilized to evaluate whether the recommended treatment plans by the system could prolong patient survival. The results indicated that the median survival time for patients who received targeted drug therapy consistent with the system’s recommendation was 205 days, whereas for those whose treatment was inconsistent with the recommendation, the median survival time was 188 days, with a statistically significant difference between the two groups (*p* < 0.05, HR = 2.050). Similarly, the median survival time for patients who received PD-1 treatment in line with the recommendation was 204 days, compared to 189 days for those whose treatment deviated from the recommendation, also showing a statistically significant difference (*p* < 0.05, HR = 1.931). Furthermore, the median survival time for patients who underwent surgery consistent with the system’s recommendation was 228 days, while for those whose surgical treatment was inconsistent with the recommendation, the median survival time was 187.5 days, revealing a highly statistically significant difference between the two groups (*p* < 0.01, HR = 2.972). These findings confirm that utilizing the Deepsurv treatment recommendation system for individualized treatment planning results in survival benefits for patients, demonstrating its potential in guiding personalized treatment strategies. (Fig. 6).

## Discussion

Since the 1960s, with the popularization of various screening methods and the progress of individualized treatment, the incidence and mortality of many common malignant tumors have declined significantly, but the incidence and mortality of pancreatic cancer are still gradually increasing, with poor overall prognosis [10, 11]. As the most common type of pancreatic cancer, early diagnosis of PDAC is an important way to improve its survival rate [12]. At present, no highly specific serum tumor marker for PDAC has been found. The most widely used serum marker in clinic is CA19-9, which is valuable for evaluating prognosis and monitoring recurrence after resection, but its positive predictive value is only 0.5–0.9% [13, 14]. The sensitivity and specificity of single serum biomarker detection are limited. Many studies have shown that the combined detection of serum markers can significantly improve the early diagnosis rate of malignant tumors [15–18].

Machine Learning (ML) is an interdisciplinary technology that integrates knowledge from multiple fields. Its core lies in leveraging algorithms to enable computers to extract patterns from vast datasets, subsequently facilitating deep analysis and research on new data samples. Since the advent of Hebbian theory in 1949, ML has undergone decades of vigorous development, particularly amidst the wave of the big data era, where novel technologies represented by deep learning have emerged endlessly, transforming ML from theoretical constructs into practical applications. Currently, ML plays an increasingly pivotal role in the medical field, particularly in oncology, pathology, and the diagnosis of certain rare diseases. Its primary methodologies encompass supervised learning, unsupervised learning, and deep learning. Among them, supervised learning is the most commonly adopted ML approach in disease diagnosis. Specific instances include the k-Nearest Neighbor (KNN) algorithm, Artificial Neural Network (ANN), Support Vector Machine (SVM) algorithm, Decision Tree (DT), Random Forest Classification (RFC) algorithm, etc. These all rely on extensive data with known labels (such as classification or diagnostic outcomes) for model training, thereby equipping machines with the ability to identify new samples and diagnose new cases. Currently, ML technologies have been employed for tumor classification and prognosis analysis, and modeling techniques based on ML algorithms have been widely used in the diagnosis and prognosis evaluation of malignant tumors and other diseases. For example, the Naresh Khuriwal team utilized an ANN model and Logistic regression model to diagnose breast cancer patients, achieving an accuracy rate of 98.5% [19]. Eiryo Kawakami et al. constructed specific predictive models for the clinical staging, typing, and prognosis of ovarian cancer based on multiple serum markers, leveraging various ML models such as GBM, SVM, RF, and Conditional RF. They found that the performance of ML models was significantly superior to traditional regression analysis, particularly in the RF model, where the accuracy rate for diagnosing benign and malignant ovarian tumors could reach 92.4%, and the accuracy rate for predicting ovarian cancer staging could also reach approximately 70% [20]. This section of the research applies ML algorithms in conjunction with routine clinical biochemical indicators and tumor markers to diagnose PDAC. The results indicate that various diagnostic models exhibit high accuracy, sensitivity, and specificity, showing a high level of consistency with the results of pathological puncture biopsy. We employed four supervised learning models: RF, NNET, SVM, and GBM, to differentiate between PDAC sample data and non-PDAC sample data. To enhance the generalization performance of the models, we incorporated data from various gastrointestinal tumors and pancreatic inflammatory diseases. The results indicate that the ML models can effectively distinguish PDAC from non-PDAC data. In the independent validation set samples, the models achieved an accuracy rate exceeding 80%, with a sensitivity rate exceeding 90%, making them promising tools for auxiliary diagnosis and screening of PDAC.

Deep Learning derived from the multi-layer neural network architecture of classical neural network technology, takes matrix data as input and generates new datasets as output through nonlinear activation mechanisms. The rise and continuous development of DL are propelling the field of machine learning towards genuine intelligence. As a frontier research area within machine learning, DL focuses on analyzing the essential patterns and manifestations of sample data, aiming to endow machines with human-like comprehension and learning capabilities. DL, a highly complex machine learning algorithm, is now widely applied in image recognition, speech recognition, and other domains. Its algorithms and hierarchical structures are extremely intricate, with commonly used algorithms including Recurrent Neural Networks (RNNs), Autoencoders, and Convolutional Neural Networks (CNNs). These technologies have been extensively utilized in natural language processing and certain medical fields, offering novel methodologies, technologies, and continuous possibilities for the advancement of artificial intelligence.

In medical research, survival models were traditionally employed to analyze the role of prognostic factors in events such as patient mortality or malignancy recurrence, thereby assisting clinicians in devising scientific treatment plans. Among these models, the CPH model is a widely accepted and recognized standard. As a semi-parametric model, CPH effectively quantifies the impact of covariates on the risk of significant events. The CPH model is based on a core assumption that the risk of death can be predicted through the linear combination of its covariates, known as the proportional hazards assumption. However, in practical applications, the risk functions of many datasets may not adhere to the linearity assumption, limiting the applicability of the CPH model. To more accurately fit the relationship between survival data and nonlinear risk functions, researchers require more diverse survival models. Given the neural networks’ advantage in handling highly complex nonlinear functions, research efforts have begun to apply neural networks for more precise modeling of nonlinear proportional hazards in actual survival datasets.

In survival modeling, neural network-based approaches primarily encompass classification methods, time-encoding methods, and the Faraggi-Simon network. The Deepsurv model adopted in this paper is a deep learning survival analysis model based on the Faraggi-Simon network. The Faraggi-Simon network notably excels in accurately predicting prognosis without relying on extensive variable feature selection. In machine learning models, when there are too many model parameters and too few training samples, the trained model is prone to overfitting. The Deepsurv model, however, can effectively mitigate overfitting through the Dropout algorithm, achieving a certain degree of regularization. Currently, the use of the DeepSurv model for prognosis research of malignant tumors mainly includes lung cancer [21–23], head and neck tumors [24], breast cancer [25], melanoma [26, 27], gastric cancer [28], and so on.

This study combined the prognosis information of patients and used regression methods to establish the CPH and Deepsurv models by incorporating the three indicators with the highest prognostic value after screening, and verified that both models were of great value for the prognostic risk assessment of PDAC, and the performance of the Deepsurv model was superior to that of the traditional CPH model. The model we established yielded a C-index of 0.722 for the training set of the CPH model, and a C-index of 0.703 for the validation set. For the Deepsurv model, the C-index was 0.738 for the training set and 0.724 for the validation set. In 2022, Keyl J et al. [29]. published a study where they constructed a random survival forest model based on clinical data from 203 patients with advanced PDAC. The model based on clinical parameters achieved a C-index of 0.71. This method outperformed the American Joint Committee on Cancer (AJCC) staging system and the modified Glasgow Prognostic Score (mGPS) in identifying high-risk and low-risk subgroups. Our models demonstrate comparable predictive abilities to the model published by this research team.

Currently, the treatment of PDAC remains a clinical challenge, with the majority of PDAC patients failing to achieve survival benefits even after receiving guideline-recommended therapies, often due to late diagnosis or ineffective treatment. Additionally, different treatment regimens yield varying therapeutic effects on patients, making it a difficult task for clinicians to select personalized treatment plans for individual patients. Employing artificial intelligence technology to predict the prognosis of different treatment options and selecting appropriate treatment measures based on these predictions represents a promising approach. This study, based on this rationale, utilizes the treatment recommendation function of Deepsurv to establish a PDAC model, which serves as a preliminary exploration of this issue. Surgical treatments such as pancreaticoduodenectomy (Whipple procedure), distal pancreatectomy, and total pancreatectomy are standard procedures recommended by the NCCN guidelines [30]. Furthermore, PD-1 immunotherapy drugs like pembrolizumab and nivolumab are also recommended by the NCCN guidelines (Category 2 A). Targeted therapies, such as nimotuzumab, anlotinib, have also been proven to significantly improve the survival of PDAC patients [31, 32]. Therefore, based on patients’ clinical treatment records, this study selects these three treatment options as categories for individualized treatment prediction. Our results indicate that patients who followed the treatment regimen recommended by our system had significantly longer survival times compared to those who did not, demonstrating the potential of the Deepsurv treatment recommendation system in guiding individualized treatment.

However, this study also has several limitations: (1) Due to the limited sample size and the lack of an independent external validation dataset, the generalization performance and practical clinical value of the model cannot be guaranteed. Although we have employed some methods to reduce the likelihood of model overfitting, such as supplementing the dataset with samples from various stages of the same patient, and using cross-validation and regularization to optimize the model, establishing an independent external validation cohort remains a task that we need to complete in our future work. (2) In the treatment of PDAC, the selection of surgery, targeted therapy, and immunotherapy should be based on a comprehensive assessment of multiple factors, including patient individual characteristics, tumor molecular subtypes, and biomarkers. For example, targeted therapies need to consider mutation sites (such as BRCA1/2 or PALB2 mutations), and immunotherapies are suitable for patients with individualized characteristics such as high microsatellite instability (MSI-H) or deficient mismatch repair (dMMR). The optimal approach is to use multi-omics data to predict treatment response. Our study only used serological markers for evaluation and prediction, which cannot achieve true individualized prediction. The treatment recommendations made using the model established in this paper are for reference only, and treatment options should still be selected in combination with gene sequencing and the relevant patient conditions.

## Conclusions

The PDAC machine learning diagnostic model constructed in this study exhibits high accuracy, sensitivity, and specificity, serving as an auxiliary diagnostic tool to enhance the precision of clinical pathological diagnosis, reduce missed and misdiagnosed cases, and minimize the use of invasive examinations. The established CPH and deep learning Deepsurv models demonstrate a high degree of consistency with the actual prognosis of PDAC patients, providing valuable reference for clinical assessment of PDAC prognosis. Patients undergoing personalized treatment guided by the Deepsurv treatment recommendation system can expect better survival outcomes.

## Data Availability

The datasets generated and/or analyzed during the current study are available from the corresponding author on reasonable request, provided that such release does not violate the privacy rights of the participants or conflict with any ethical or legal obligations. All materials used in this study, including experimental protocols, questionnaires, and analysis scripts, are likewise available for sharing upon request, subject to the same conditions. The authors encourage collaboration and data sharing within the scientific community to advance knowledge and understanding in the field.
